# Molecular surveillance of artemisinin resistance falciparum malaria among migrant goldmine workers in Myanmar

**DOI:** 10.1186/s12936-017-1753-8

**Published:** 2017-03-01

**Authors:** Myat Htut Nyunt, Bo Wang, Khin Myo Aye, Kyin Hla Aye, Jin-Hee Han, Seong-Kyun Lee, Kay Thwe Han, Ye Htut, Eun-Taek Han

**Affiliations:** 1 Department of Medical Environmental Biology and Tropical Medicine, School of Medicine, Kangwon National University, Chuncheon, Republic of Korea; 2 Department of Medical Research, Yangon, Myanmar; 3 Department of Clinical Laboratory, The First Affiliated Hospital of Anhui Medical University, Anhui, People’s Republic of China

**Keywords:** Malaria, Artemisinin resistance, Migrant, Myanmar, Molecular surveillance

## Abstract

**Background:**

Artemisinin resistance has been reported in Greater Mekong Sub-region countries, including Myanmar. After discovery of artemisinin resistance marker (K13), molecular surveillance on artemisinin resistance in endemic regions have been conducted. As the migrant population represents a high percentage of malaria cases, molecular surveillance of artemisinin resistance among migrant workers is of great concern.

**Methods:**

A cross-sectional survey was conducted in Shwegyin Township, where migrants work in the goldmines. Blood samples were collected from uncomplicated *Plasmodium falciparum*-infected migrant workers by active and passive cases screening with rapid diagnostic testing (RDT) and microscopy. Amplification and sequence analysis of artemisinin resistance molecular markers, such as *k13*, *pfarps10*, *pffd*, *pfmdr2*, *pfmrp1*, *pfrad5*, and *pfcnbp*, were carried out and *pfmdr1* copy number analysis was conducted by real-time PCR.

**Results:**

Among the 100 *falciparum*-infected patients, most were male (90%), of working age (20–40 years) with median parasite density of 11,166 parasites/µL (range 270–110,472 parasites/µL). Artemisinin resistance molecular marker, *k13* mutations were detected in (21/100, 21.0%) in which composed of a validated marker, C580Y (9/21, 42.9%) and candidate markers such as P574L (5/21, 23.8%), P667T (5/21, 23.8%) and M476I (2/21, 9.5%). Underlying genetic markers predisposing to become *k13* mutants were found as V127M of *pfarps10* (41/100, 41.0%), D153Y of *pffd* (64/100, 64.0%), T484I of *pfmdr2* (58/100, 58.0%) and F1390I of *pfmrp1* (24/100, 24.0%). The *pfmdr1* copy number analysis revealed six copy numbers (1/100, 1.0%), three (2/100, 2.0%), two (8/100, 8.0%) and only one copy number (89/100, 89.0%). Only one sample showed both *k13* mutation (P667T) and multiple copy number of *pfmdr1*.

**Conclusions:**

High mutant rate of artemisinin resistance markers and relatively high *pfmdr1* copy number among isolates collected from migrant goldmine workers alert the importance of containment measures among this target population. Clinical and molecular surveillance of artemisinin resistance among migrants should be scaled up.

**Electronic supplementary material:**

The online version of this article (doi:10.1186/s12936-017-1753-8) contains supplementary material, which is available to authorized users.

## Background

Artemisinin-based combination therapy (ACT) is the most effective anti-malarial treatment for falciparum malaria [1]. However, artemisinin resistance was reported in clinical studies in Cambodia as early as 2006 [2], followed by other Southeast Asian counties, including Myanmar, Laos, Thailand, and Vietnam [3]. In Myanmar, delayed clearance of the parasite after treatment with ACT has been reported since 2009 in southern Myanmar [2] and reduced susceptibility of artesunate was observed in 2010 [4]. In Myanmar, a multifaceted artemisinin resistance containment programme was initiated in 2011 [5] and, according to the global plan for artemisinin resistance containment (GPARC), artemisinin resistance zones were categorized as Tier I: where there was evidence of artemisinin resistance; Tier II: where there was suspected evidence of artemisinin resistance; and Tier III: the remaining malaria prevalence areas [6]. According to the MARC programme, migrant and mobile populations are highly vulnerable and the potential spreader of resistant parasites [7].

The worldwide burden of malaria has been decreasing significantly and the number of countries moving to malarial elimination is increasing [8]. The World Health Organization (WHO) has initiated a strategy for malaria elimination in the Greater Mekong Sub-region by 2030 [9]. Drug resistance is one of the challenges to successfully achieving this goal [10]. Surveillance of artemisinin resistance along with appropriate action to eliminate resistant strains is important in successfully containment of the resistant parasite. After the discovery of artemisinin resistance molecular markers (*k13*), worldwide prevalence of these markers was documented [11, 12]. Other markers, such as *pfmrp1* (multidrug resistance protein 1 gene), *pfcnbp* (cyclic nucleotide-binding protein) and *pfrad5* (DNA repair protein RAD5 homologue) were also identified as potential drug resistance markers [13]. A genome-wide association study reported that *pfarps10* (apicoplast ribosomal protein S10), *pffd* (ferredoxin), *pfmdr2* (multidrug resistance protein 2) and *pfcrt* (chloroquine resistance transporter) gene mutations are significantly associated with delayed clearance of parasites after ACT, indicating the underlying genetic background for artemisinin resistance [14].

Migrant and mobile populations are a major concern for malaria transmission and are a target group for the artemisinin containment programme implemented in Myanmar, Laos, Cambodia, Thailand, and Vietnam [15, 16]. Because of the nature of these populations, it is difficult to conduct regular longitudinal monitoring and surveillance of the occurrence of malaria in these groups. In this study, molecular marker analysis was carried out in one gold mining area in Myanmar to assess the status and distribution of artemisinin resistance.

## Methods

### Study design and study population

**Fig. 1:**
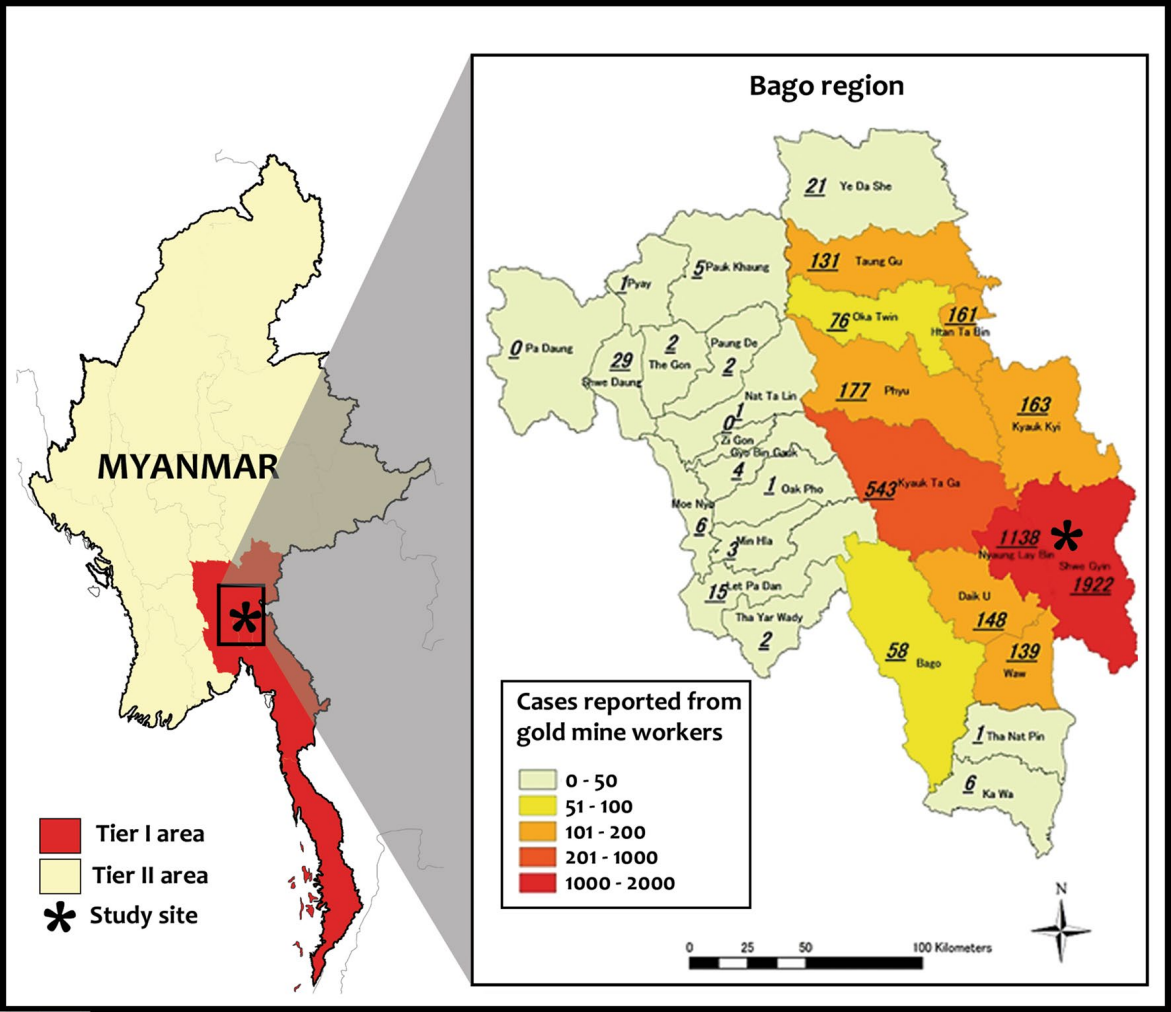
Map of the Bago Region showing the numbers of cases of falciparum malaria diagnosed within each administrative township who reported that they were migrant goldmine workers. The study site, Shwegyin showed the highest reported number of cases of malaria from migrant goldmine workers than from neighbouring areas as of 2010. It is one of the Tier I areas of Myanmar’s artemisinin resistance containment zone

**Fig. 2:**
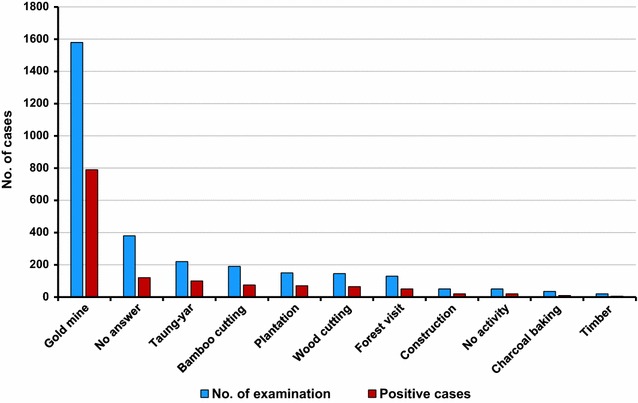
Occupation of the examined cases and malaria cases in active cases detection. Data were retrieved from the township active case detection [14] report conducted in 2010. Goldmine workers were the majority of malaria cases in this study site. Taung-yar: slash-and-burn cultivation

This cross-sectional analysis study was conducted in Shwegyin (22°20′0″ N, 95°56′0″ E), one township of the Myanmar artemisinin resistance containment (MARC) zone (Fig. 1). According to the 2014 census, a population of 107,462 was living in 2440.1 sq km. As the Shwegyin area is famous for gold mining, migrant and mobile populations are working in the deep forest goldmine and bearing a high burden of malaria as compared to the neighbour township (Fig. 1). Moreover, the majority of reported malaria cases come from the migrant population in Shwegyin (Fig. 2) [17].

Uncomplicated *Plasmodium falciparum*-infected samples from migrant goldminers or their family members were collected from Shwegyin during 2013–2015. Active and passive cases detection was carried out for sample collection. All malaria suspected cases were initially screened by rapid diagnostic testing (RDT) followed by microscopy examination of peripheral blood smear. The patients with parasite count more than 500/µL were included in the study although all positive cases were treated according to the national malaria treatment guideline. Whole blood samples were collected in EDTA-coated tubes for further molecular analysis.

### DNA extraction, amplification and sequencing

Parasite DNA was extracted from the whole blood using QIAamp DNA Blood Mini Kit (QIAGEN) according to manufacturer’s instruction. A nested PCR approach was designated to amplify target genes using a specific pair of primers (Additional file 1). Amplifications were performed in a reaction mixture that contained 0.25 mM of each dNTP, 10 mM Tris–HCl (pH 9.0), 30 mM KCl, 1.5 mM MgCl_2_, 1.0 units of *Taq* polymerase (Bioneer, Seoul, Korea), 0.02 µM primers, and 2 µl of genomic DNA. For target gene amplifications, initial denaturation at 95 °C for 5 min was followed by 35 cycles of 95 °C for 30 s, 58 °C for 1 min (*k13*, *pfarps10*, *pfmdr2*) or 62 °C for 1 min (*pffd*), 72 °C for 1.5 min, and a final extension of 72 °C for 10 min. Using 1 µL of the nested-1 product as a template, the same conditions were applied for the nested-2 PCR except for an annealing temperature of 60 °C for 1 min (*k13*, *pffd*, *pfmdr2*) or 62 °C for 1 min (*pfarps10*) and 72 °C for 1 min with 30 cycles.

For *pfmrp1, pfcnbp* and *pfrad5* gene amplification, PCR was performed with an initial denaturation at 94 °C for 5 min and 30 cycles at 94 °C for 30 s, 56 °C for 30 s, and 72 °C for 1 min. After amplification of the targets, PCR clean-up was carried out using MEGAquick-spin DNA fragment Kit purification (iNtRON, Republic of Korea) following manufacturer’s instruction. Purified PCR products were sequenced directly by using each primer for target gene amplification. The deduced amino acid sequences were aligned and analysed with the Lasergene^®^ software (DNASTAR, Madison, WI, USA) using the reference sequences of 3D7 retrieved from Plasmodium data base [18]. The sequence data from this study have been submitted to GenBank under accession numbers KJ956790–KJ956797 and KY195978–195988.

### Real-time PCR for copy number analysis of the *pfmdr1* gene

Copy number analysis on *pfmdr1* genes was carried out according to procedures described previously [19, 20]. Briefly, each 20 μL contained 10 μL of multiplex PCR ROX master mix, 0.3 µM of primers, 0.2 µM of probe, 0.3 µM of forward and reverse β-tubulin gene and 2 µL of template DNA. In each run of real-time PCR, 3D7 and Dd2 strain were used as known control for copy number analysis. The thermal cycling conditions were 95 °C for 5 min and then 40 cycles of 95 °C for 15 s and 58 °C for 1 min by ABI 7500 (Applied Biosystems, Foster City, CA, USA).

### Statistical analysis

All data were double checked and analysed by Microsoft Excel and SPSS (Version 22.0. Armonk, NY: IBM Corp). Prevalence of each molecular markers was separately and combined analysed as percentage. Age group, sex and parasitaemia were analysed for association on prevalence of each molecular markers by Chi square test with 95% confidence interval. Multiple and single copy number of *pfmdr1* genes and *k13* mutations were analysed with co-occurrence of other molecular markers.

### Ethical approval and consent to participate

The study was conducted after receiving ethical approval from the institutional ethical committee, Department of Medical Research, Myanmar (Approval no. 52/ethic/2012). Written consent was taken from all participants. Participation in this study was entirely voluntary.

## Results

A total of 100 *P. falciparum* positive samples were included in this study, and as collection was migrant populations in gold mining areas, most were from men of working age. The median parasite density was 11,166 parasites/µL (range 270–110,472 parasites/µL). The basic demographic characteristics of the participants are shown in Table 1.

**Table 1:** Basic demographic characteristics of the participants

Total participants (n)	100
Male: female	9:1
Median age (range)	23
Working age group (18–50 years)	78
Median parasite density (range) (parasites/µL)	11,166 (270–110,472)

All of the samples were well amplified for all the molecular markers. Kelch gene, *k13* (PF3D7_1343700) sequences analysis revealed that 21/100 (21%) samples were non-synonymous mutation after amino acid 440. There were only four mutation sites were observed. Among them, C580Y accounted for (9/21, 42.9%), P667T (5/21, 23.8%), P574L (5/21, 23.8%) and M476I (2/21, 9.5%).

Similarly, V127M mutation of *pfarps10* (PF3D7_1460900.1) accounted for 41/100 (41.0%), D153Y mutation of *pffd* (PF3D7_1318100) for (64/100, 64.0%), T484I of *pfmdr2* (PF3D7_1447900) for (58/100, 58.0%), F1390I of *pfmrp1* (PF3D7_0112200) for (24/100, 24.0%), S1188L of *pfcnbp* (PF3D7_1417400) for (17/100, 17.0%), S1158A of *pfrad5* (PF3D7_1343400) for (46/100, 46.0%), and N1131I of *pfrad5* for (7/100, 7.0%) (Table 2).

**Table 2:** Summary of the single nucleotide polymorphisms (SNPs) of the molecular markers

Target genes	SNPs	Codon position	Amino acid (nucleotide) sequence		No. of isolates/total no. of cases
			Reference	Mutant	
*k13*	C580Y	580	C (T**G**T)	Y (T**A**T)	9/100
	P574L	574	P (C**C**T)	L (C**T**T)	5/100
	P667T	667	P (**C**CA)	T (**A**CA)	5/100
	M476I	476	M (AT**G)**	I (AT**A)**	2/100
*pfarps10*	V127M	127	V (**G**TG)	M (**A**TG)	41/100
*pffd*	D153Y	153	D (**G**AC)	Y (**T**AC)	64/100
*pfmdr2*	T484I	484	T (A**C**A)	I (A**T**A)	58/100
*pfmrp1*	F1390I	1390	F (**T**TT)	I (**A**TT)	24/100
*pfcnbp*	S1188L	1188	S (T**C**G)	L (T**T**G)	17/100
*pfrad5*	N1131I	1131	N (A**A**T)	I (A**T**T)	7/100
	S1158A	1158	S (**T**CA)	A (**G**CA)	46/100

All of the non-synonymous mutations are listed and the changes of nucleotide from reference sequences (3D7) are shown in bold

**Fig. 3:**
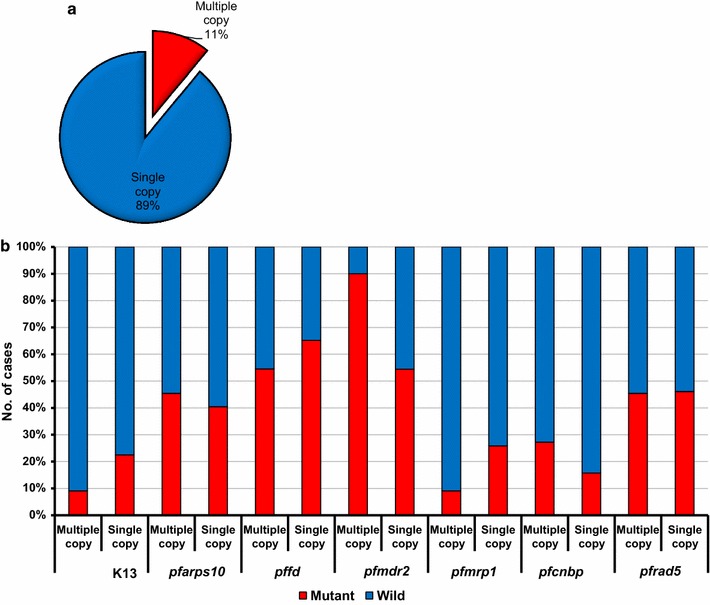
Co-occurrence of molecular markers among *pfmdr1* multiple and single copy number. The distribution of single and multiple copy number of *pfmdr1* gene (**a**) and co-occurrence of other molecular markers among single and multiple copy number of *pfmdr1* gene (**b**)

*Pfmdr1* copy number analysis showed that 89/100, 89% were single copy number and overall mean copy number of 1.296. Among the multiple copy number samples, eight samples were two copies, two samples were three copies and one sample was six copy number. There was no correlation on mutations of the molecular markers or multiple copy numbers of the *pfmdr1* gene with the age group (p = 0.5310), sex (p = 0.2911) and hyperparasitaemia (p = 0.2311). Based on the co-occurrence of the different mutations among seven markers and *pfmdr1* copy number, 54 isotypes were observed (Additional file 2). Only three samples showed no mutation with single copy number of *pfmdr1*. An additional 11 samples were observed that had only one mutation of molecular markers. Among the multiple copy number of *pfmdr1* samples, T484I of *pfmdr2* mutation showed 9/11 (81.8%), D153Y of *pffd* found as 6/11 (54.4%) and *pfrad5* accounted for 5/11 (45.5%). A significantly higher mutant rate of *pfmdr2* was observed among the *pfmdr1* multiple copy number (p = 0.0307) (Fig. 3).

**Fig. 4:**
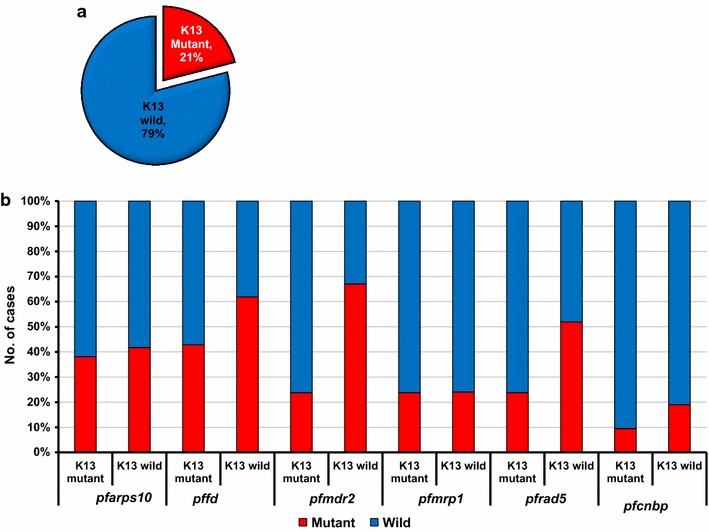
Co-occurrence of the molecular markers among *k13* kelch mutant and wild type. Distribution of *k13* wild and mutant (**a**) and co-occurrence of other molecular markers among the *k13* mutants and wild-type alleles (**b**)

As *k13* gene mutation is a well-known marker for artemisinin resistance, co-occurrence of *k13* mutants and other marker mutants were also assessed. Among the 21 samples of *k13* mutants, D153Y of *pffd* (9/21, 42.9%), V127M of *pfarps* (8/21, 38.1%), T484I of *pfmdr2* (5/21, 23.8%), F1390I of *pfmrp1* (5/21, 23.8%), S1158A of *pfrad5* (5/21, 23.8%) and S1188L of *pfcnbp* (2/21, 9.5%) were observed. The *pfmdr2* and *pfrad5* mutations were at a higher mutant rate among *k13* wild-type samples (p = 0.0004 and p = 0.0217, respectively (Fig. 4).

## Discussion

In this study, a total of 100 uncomplicated falciparum malaria patients from migrant goldminers were included for molecular analysis. Well-known artemisinin resistance molecular marker, *k13* genes as well as other markers, including *pfarps10, pffd, pfmdr2, pfmrp1, pfcnbp*, and *pfrad5* genes were analysed. Moreover, to estimate mefloquine or lumefantrine resistance, *pfmdr1* copy numbers were assessed.

Currently, *k13* is the only molecular markers confirmed by transfection and clinical data [11, 21]. Although more than one hundred of *k13* mutations were reported, non-synonymous mutations after amino acid position 440 were found to be linked with artemisinin resistance [21, 22]. In this study, approximately one-fifth of the samples (21%) showed four non-synonymous mutations in *k13* genes. Among them, C580Y is predominant (9/21, 42.86%) followed by P574L (5/21, 23.81%), P667T (5/21, 23.81%) and M476I (2/21, 9.523%). The C580Y, a validated marker for artemisinin resistance has been observed in falciparum endemic countries in South–east Asia region [22, 23]. In Myanmar, C580Y mutation was observed in 11% of the samples in Kawthaung, southern Myanmar. Although it was also reported in Kayin State, Myanmar–Thai border area [24], previous survey in Shwegyin showed no C580Y mutation [25]. Meanwhile, it has been increasing in Thai side of the border area [23]. In this study, C580Y was detected as 9/21 (42.9%) of all non-synonymous mutations in K13 propeller region and might be increasing in prevalence, although the overall proportion of isolates with mutations in the propeller region does not appear to have changed compared to the previous study conducted in the same study site [26].

Although P667T and M476I were not common *k13* mutations, they were also reported as associated markers with delayed parasite clearance after treatment with ACT in Thailand [27] and Cambodia [11]. Interestingly, in this study, P667T was the first reported *k13* mutation in Myanmar, although it was observed as one of the markers showing delayed parasite clearance in Thailand [23].

According to the genome-wide association study (GWAS) [14], other markers, such as *pfarps10*, *pffd, pfmdr2*, and *pfcrt* also indicate a genetic background predisposing to become *k13* mutant. Moreover, these markers showed equal geographical distribution of *k13* mutations [14]. In this study, D153Y of *pffd* showed the highest mutant rate that accounted for 64%, followed by T484I of *pfmdr2* (58%) and V127M of *pfarps10* (41%), reflecting similar findings by GWAS at the same study site. Moreover, these markers were also reported in asymptomatic infections among the local residents of this study site [28]. However, there is no enough evidence on the interpretation and mechanism of these markers.

Before discovery of K13 molecular marker, *pfmrp1*, *pfcnbp* and *pfrad5* were identified as potential markers associated with artemisinin resistance. Among them, *pfmrp1* and *pfcnbp* are denoted as the markers of less certain evidence. The F1980I mutation of *pfmrp1* was found as an artemether–lumefantrine combination, reflecting multi-drug resistance status, especially for lumefantrine [29, 30]. However, no mutations in *pfmrp1* that showed the delayed clearance of parasite was identified in GWAS. It may be because of the phenotype used in GWAS and the markers are unlikely to be of relevance to artemisinin resistance in Southeast Asia region [14]. Meanwhile, *pfcnbp* and *pfrad5* were reported as potential candidates to assess delayed parasite clearance of parasites as these two proteins are involved in post-replication repair process [13]. However, *pfcnbp* is a gene annotated as a pseudogene [13] and it did not appear as an important markers related with delayed parasite clearance in GWAS in South–east Asia region [14].

The marker that are likely to have been associated with artemisinin resistance only because of the linkage disequilibrium include *pfrad5*. It is important to understand that mutations in *pfrad5* was almost certainly associated with artemisinin resistance [13] because of the linkage to the C580Y of Cambodia isolates in *k13* genes that was close to *pfrad5* on chromosome 13. In this study, 46.0, 24.0 and 17.0% were noted as non-synonymous mutations in *pfrad5, pfmrp1* and *pfcnbp*. However, the *k13* mutation in Myanmar has a different genetic background to that in Cambodia and it is unable to interpret directly based on these mutations in *pfrad5, pfmrp1* and *pfcnbp* in Myanmar.

The *pfmdr1* multiple copy number also linked to resistance to ACT, especially for combinational therapy with mefloquine [31]. Both *k13* mutations and *pfmdr1* multiple copy in clinical samples increased risk of treatment failure by up to 14 times [23]. In this study, *pfmdr1* multiple copy was observed in 11% of the samples, which was higher than a previous multi-site study in Myanmar [20], but similar to a multi-countries study conducted in Southeast Asia region [32] and lower than a study conducted in Thailand [23]. Among the *pfmdr1* multiple copy number samples, eight were two copies, two were three copies and one sample showed six copies. Only one sample showed both *k13* and *pfmdr1* multiple copy number. Moreover, only *pfmdr2* (T484I) showed the significantly higher mutant rate in *pfmdr1* copy number, reflecting potential partner drug resistance.

## Conclusions

In this study, artemisinin resistance molecular markers among falciparum-infected samples from migrant goldminers were first reported. There are very few studies focusing on drug resistance in migrant populations although they have been recognized as a target population in containment programmes. High mutant rate of artemisinin resistance molecular marker, *k13* in migrant goldmine workers alert to emphasize the surveillance on artemisinin resistance in this target group population. Although high co-occurrence of *k13* and *pfmdr1* copy number was not observed, a significant 11% of samples with multiple copy number of *pfmdr1* showed potential partner drug resistance. Scaling-up of clinical and molecular surveillance of migrant and mobile populations should be emphasized in the strategy to eliminate artemisinin resistance in the Greater Mekong Sub-region.

## Additional file

**Additional file 1.** Paris of primers used to amplified the target genes.

**Additional file 2.** Isotypes based on co-occurance of the different mutations among seven markers and *Pfmdr1* copy number
