# Supplementary material for: Molecular surveillance of artemisinin resistance falciparum malaria among migrant goldmine workers in Myanmar
Source: Malar J. 2017 Mar 1;16:97. doi: 10.1186/s12936-017-1753-8 (PMC5333451; doi:10.1186/s12936-017-1753-8)
Supplement: Supplementary file 1 — Additional file 1. Paris of primers used to amplified the target genes. [file 12936_2017_1753_MOESM1_ESM.docx]

**Additional file 1. Primers used to amplify the molecular markers**

| **Target gene** | **PCR reaction** | **Primer name** | **Primer sequences (5'–>3')** |
| --- | --- | --- | --- |
| K13 (PF3D7_1343700) | Primary | K13_1R | CGG AGT GAC CAA ATC TGG GA |
|  |  | K13_3NF | AGC GGA AGT AGT AGC GAG AA |
|  | Nested | K13_2F | GCC AAG CTG CCA TTC ATT TG |
|  |  | K13_3R | GCC TTG TTG AAA GAA GCA GA |
| *pfarps10* (PF3D7_1460900.1) | Primary | PfARPS_F3 | TGC GAC TTT TAG GGT GTG GA |
|  |  | PfARPS_N2R | CAT GGT ACC ACT TTT TCT TTT CCA |
|  | Nested | PfARPS_F1 | TTG TAG CAG GCC CAA TTC CC |
|  |  | PfARPS_N2R2 | TCT GGG TAA TTT GAC ATT CAT |
| *pffd* (PF3D7_1318100) | Primary | PfFD_F1 | AGT TGT TCT ACA TGC GCA GC |
|  |  | PfFD_R1 | AAT GTG CGC TTG TAG TGC AT |
|  | Nested | PfFD_F2 | TGC GCA GCA AAA TTA GTC GAA |
|  |  | PfFD_R2 | CAT TCC CCA TTT CAA TCA TAT CCA |
| *pfmdr2* (PF3D7_1447900) | Primary | Pfmdr2_F1 | TTT GTG GCC AAG CAA AAG GA |
|  |  | Pfmdr2_R1 | TCT TTG TCG TTC TCC TCC TGA |
|  | Nested | Pfmdr2_F8 | AGA GGT ACC GAG AGT GCT AA |
|  |  | Pfmdr2_R8 | AGA GCA CAT GTT GTA CCT GGT T |
| *pfmrp1* (PF3D7_0112200) | Primary | Pfmrp1-F | TGTAACATC TAT AGT AAT GCA TTG TCT GG |
|  |  | Pfmrp1-R | TTG TGT TGT TAT TAC ATT TAA TTC ATT TTC |
| *pfrad5* (PF3D7_1343400) | Primary | Pfrad5_F | GAC GTG AAG AAG GTA TTT AAA AGT GAA |
|  |  | Pfrad5_R | GAA ATA ATC GTC TGC ACA TTT TTT AC |
| *pfcnbp*  (PF3D7_1417400) | Primary | Pfcnbp_F | GAA GAA AGT ATA AGT AAA ACT AAA AAT GAA |
|  |  | Pfcnbp_R | TTC CTG TTT CAT TAT TTG CTT TC |
